# Acute kidney injury and nephrotic syndrome caused by a “magic pill” 

**DOI:** 10.5414/CNCS111804

**Published:** 2025-09-04

**Authors:** Yangming Cao, Thao Phan, Patil Armenian, Jonathan E. Zuckerman

**Affiliations:** 1 Division of Nephrology, Department of Internal Medicine, UCSF Fresno,; 2 The Nephrology Group, Fresno,; 3 Department of Internal Medicine, Clovis Community Medical Center, Clovis,; 4 Department of Emergency Medicine, UCSF Fresno, Fresno, CA, and; 5 Department of Pathology and Laboratory Medicine, David Geffen School of Medicine at the University of California Los Angeles, Los Angeles, CA, USA

**Keywords:** diclofenac, NSAID, acute interstitial nephritis, nephrotic syndrome, IgA nephropathy

## Abstract

Introduction: The sudden onset of nephrotic syndrome (NS) and acute interstitial nephritis (AIN) seems to be an uncommon but distinct nonsteroidal anti-inflammatory drug (NSAID)-related renal syndrome. Case presentation: We present such a case in a patient who took a “magic pill” for gout. Renal biopsy revealed minimal change disease (MCD), acute interstitial nephritis (AIN), severe acute tubular injury (ATI), and IgA nephropathy (IgAN). He was treated with an aborted course of high-dose prednisone, with complete resolution of his renal diseases. The pathologic finding of the combination of MCD and AIN raised the possibility of a drug effect. One of the pills was analyzed and found to be primarily composed of diclofenac. Initially, we considered IgAN a bystander, considering primary IgAN is the most common glomerulonephritis worldwide, especially in Asians and Hispanics. However, the complete resolution of urinary findings after discontinuation of the pill followed by a few days’ treatment with prednisone, together with no recurrence of the kidney disease over 6 years, made us speculate that IgAN may have also been triggered by diclofenac. Conclusion: We presented a case of AIN, MCD, and IgAN associated with diclofenac masquerading as a “herbal” medicine. The cause was suggested by pathology and confirmed with high-resolution liquid chromatography mass spectrometry testing of the pills. A history of NSAID use should be diligently sought in any patient who presents with NS and AIN. In addition, this is the first report of IgAN possibly induced by NSAID without recurrence after 6 years’ follow-up.

Note: An abstract of this case was presented at North American Congress of Clinical Toxicology, September 10, 2020; Virtual conference. 

## Introduction 

The combination of nephrotic syndrome (NS) and acute interstitial nephritis (AIN) seems to be an uncommon but distinct nonsteroidal anti-inflammatory drug (NSAID)-related renal syndrome [1]. We presented such a case in a patient who took a “magic pill” for gout. Renal biopsy revealed the combination of minimal change disease (MCD) and AIN, which suggested a possible drug-related effect. One of the pills was submitted for analysis and was, indeed, found to be primarily composed of diclofenac. In addition, renal biopsy found coexisting IgA nephropathy (IgAN) which was initially considered a bystander primary IgAN. However his long-term clinical course suggested his IgAN was related to diclofenac. To our best knowledge, this is thus far the first report of IgAN associated with NSAID use. 

## Case presentation 

**Figure 1.**
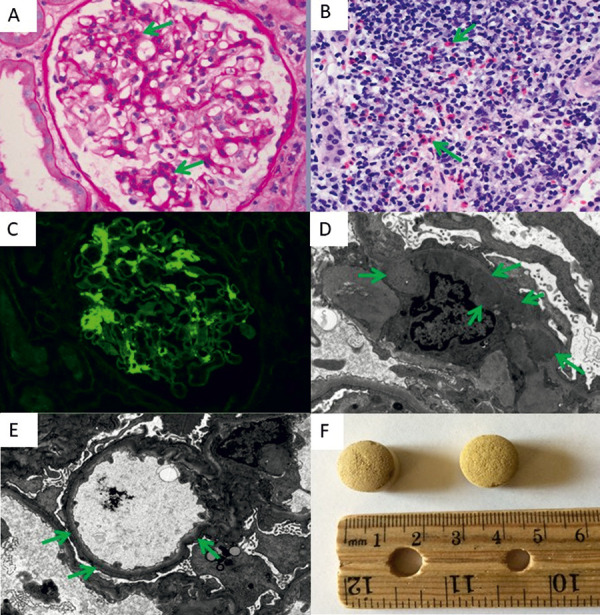
Kidney biopsy (A – E): light microscopy with periodic acid–Schiff stain (A), hematoxylin-eosin stain (B, arrows denote eosinophils), immunofluorescence with IgA (C), transmission electron microscopy (D, original magnification × 6,800, and E, original magnification × 4,800). Arrows denote the typical areas of interest in each panel, respectively. The “magic pills” (F).

On July 31, 2018, a 45-year-old Hmong male presented to our emergency department (ED) with nausea, dizziness, and ankle swelling for 1 week. His appetite was fair and he drank plenty of fluids. In ED he was found to have elevated serum blood urea nitrogen (BUN) (54 mg/dL) and creatinine (3.1 mg/dL). Renal function did not improve after he was given 1 L normal saline intravenously. He had been urinating regularly with normal urine color though the urine seemed foamy lately. He might have had blood test done 2 years prior, but his primary care physician had retired, and he had never been told of any kidney problems. His past medical history was significant only for gout. Upon question, he admitted taking an “herbal pill” for gout attack as needed. He stated: “when I take it, it really works. It is a magic pill”. He bought and took a total of ~ 100 pills in the past year. He took one pill b.i.d. for gout attack recently. The pills were bought from a woman on a street corner of his neighborhood. Neither the pills nor the small plastic bag holding the pills had any labeling. He worked as a hairdresser. Physical exam revealed normal vital signs and 1+ leg edema bilaterally. Laboratory tests showed normal complete blood count with differential including eosinophils, low serum albumin (1.7 g/dL) and high cholesterols. Urinalysis showed protein > 500 mg/dL, no blood, RBC 2/HPF and WBC 7 /HPF. The 24-hour urine protein was 19,346 mg with a protein/creatinine ratio of 19.04 g/g (Table 1). He was admitted for (presumed) non-oliguric acute kidney injury (AKI) and NS. Renal ultrasound revealed normal-sized kidneys, and echocardiogram was normal. CT-guided renal biopsy was done the next day, but the report was not available until shortly after discharge (Figure 1). Over the next few days, the following tests came back as normal or negative: CK, TSH, hepatitis B and C, HIV, RPR, cryoglobulin, ANA, C3, C4, dsDNA, SSA, SSB, rheumatoid factor, antiphospholipid antibodies, anti-cardiolipin antibodies, ANCA, anti-GBM antibody and PLA2R antibody. Serum protein electrophoresis showed one or more fractions were outside the reference interval with serum free light chain ratio of 1.67, but serum protein immunofixation electrophoresis was normal. 

Light microscopy of renal biopsy showed mild segmental to global mesangial hypercellularity (Figure 1A). Focal segmental endocapillary hypercellularity was also present. There was diffuse and focally heavy interstitial inflammation composed of lymphocytes, histiocytes, plasma cells, and conspicuous eosinophils (Figure 1B). Tubules exhibited diffuse loss of brush border staining, epithelial cell flattening, and mitotic figures. There was ~ 5% interstitial fibrosis / tubular atrophy and mild arteriosclerosis. Segmental sclerosis, necrotizing lesions, crescents, vasculitis, and thromboses were not present. By immunofluorescence microscopy, mesangial regions exhibited diffuse segmental to global granular staining with IgA (4+), IgM (1+), C3 (2+), and κ (2 – 3 +) and λ (2 – 3+) (Figure 1C). Electron microscopy demonstrated prominent finely granular paramesangial electron-dense (immune complex) deposits in mesangial regions (Figure 1D). Podocytes exhibited near complete foot process effacement (> 80% of surface areas) (Figure 1E). These findings were consistent with MCD, AIN, severe acute tubular injury (ATI), and IgAN with Oxford score of M1E1S0T0C0 [2]. The combination of MCD and AIN raises the possibility that both processes may be related to a drug effect such as from NSAIDs. A few months later, the patient agreed to provide the “magic pills” for test. The pills had a coarse surface with yellowish color and measured 1 cm in diameter, without any imprint or markings (Figure 1F). One of the pills was analyzed with high-resolution liquid chromatography mass spectrometry and found to be primarily composed of diclofenac. There were no true herbal components detected. 

His serum creatinine worsened to 4.5 mg/dL by the time of discharge on August 3, 2018. He was empirically treated with prednisone 60 mg (0.8 mg/kg) PO q.d. for 2 days in hospital and was to continue it at home. However, he did not pick up the prescription until seen in the nephrology office on August 6, 2018. At that point, he started taking prednisone 60 mg PO q.d., but 7 days later the dose had to be reduced to 40 mg PO q.d. due to blurred vision. He stopped prednisone 2 days afterwards due to persistent blurred vision. At follow-up, he quickly improved with resolution of leg edema and blurred vision, and his renal function and urinary findings normalized over the following 1 month (Table 1). He was advised to refrain from taking any NSAIDs. Renal function and urine tests remained normal every 2 years up to July 2024. 

## Discussion and conclusions 

Although the differential diagnosis for each of the pathologic diagnoses is beyond the scope of this discussion, the combination of MCD and AIN raises the possibility that both processes may be related to a drug effect. The sudden onset of NS and AIN seems to be an uncommon but distinct NSAID-related renal syndrome [1]. The NS was explained by MCD. AKI was explained by AIN and ATI, while ATI could be seen in the setting of NS alone. IgAN was considered a bystander but it showed two of the poor prognostic factors (diffuse mesangial hypercellularity and focal mild endocapillary hypercellularity), and as such clinical follow-up was recommended. Hair dressers are exposed to chemicals such as formaldehyde and glyoxylate which both can cause AKI by mechanisms which are different from AIN and MCD. Both mercury and p-phenylenediamine in hair dye were associated with membranous nephropathy (MN) in case reports [3, 4]. The lack of recent exposure to new chemicals and the later clinical course excluded his occupation as a risk because of no recurrence despite his continuing his job. 

Before the hospitalization, he did not take allopurinol or colchicine. To rule out any possibility that he failed to tell us any other medicines that he was taking and thus a possible cause of nephritis, we, at the time of writing this article, reached out to his pharmacies for the dispensary history. He has filled allopurinol and colchicine occasionally in the past few years up to 2024. This excluded these medicines as a cause of nephritis because of no disease recurrence while taking these medicines. In August 2023, when he was admitted for left ankle gouty tophus complicated by abscess after he poked the tophus with a needle, he did not have nephritis flare-up. This ruled out gouty attack as a cause of his nephritis. He has no other secondary causes of IgAN such as chronic liver disease, inflammatory bowel disease, ankylosing spondylitis, HIV infection, or anti-tumor necrosis factor-α therapy. The identification of diclofenac in the pills and the exclusion of other causes substantiated our suspicion of NSAID as the cause of AIN. A history of NSAID use should be diligently sought in any patient who presents with AIN and nephrotic syndrome. Pills that may be listed as “herbal” may contain dangerous pharmaceuticals. Lack of regulation in the production process of these medications as well as limited user insight may result in potentially harmful and life-threatening consequences. 

There are no treatment guidelines for AIN. The first ongoing randomized trial (PRAISE) may shed lights on this [5]. Therefore discontinuation of the potential causative agent remains a mainstay of therapy for drug-induced AIN. Glucocorticoids, despite controversy, are frequently used in the treatment of AIN with variable effects. A retrospective study suggested steroids, if administered, should be started promptly (within 14 days) after diagnosis of drug-induced AIN to avoid subsequent interstitial fibrosis and an incomplete recovery of renal function [6]. Nevertheless, NSAID-induced AIN generally does not respond to glucocorticoids. The case reports of diclofenac-induced AIN with or without nephrotic syndrome, by searching Pubmed for English literature using diclofenac and acute interstitial nephritis as keywords, are summarized in Table 2 [7, 8, 9, 10, 11, 12]. Some of these patients were treated for a prolonged period of up to 6 months. All of the patients, with or without steroid treatment, had complete resolution of nephritis except 2 who had partial recovery and were left with chronic kidney disease. It is hard to interpret the role of the aborted course of high-dose prednisone in our patient. It might have some effect on AIN and MCD, but likely not on IgAN if it was primary IgAN. 

Initially we considered IgAN a bystander, considering primary IgAN is the most common glomerulonephritis worldwide, especially in Asians and Hispanics. However, the complete resolution of urinary findings after discontinuation of the pill followed by a few days’ treatment with prednisone, together with no recurrence of the kidney disease over 6 years, makes us take a second thought. In the majority of patients, primary IgAN takes a slow but relentless clinical course. In a large IgAN cohort, 50% of patients reached kidney failure or died over a median follow-up of 5.9 years [13]. Even patients traditionally regarded as being low risk, with proteinuria < 0.88 g/g, had high rates of kidney failure within 10 years [13]. Although supportive, immunosuppressive-based therapies constitute standard clinical practice, no effective and disease-specific therapies were available until recently. The long-term effect of the newly approved drugs (budesonide, sparsentan, and iptacopan) for primary IgAN remains to be seen. The striking IgA immunofluorescent staining and abundant immune complex deposits in the biopsy indicated his IgAN was robustly active. Therefore the almost immediate, complete, and long-lasting clinical resolution was beyond expectations. He might have had underlying, indolent primary IgAN, exacerbated by diclofenac, but more likely, he developed new-onset secondary IgAN triggered by diclofenac. Lack of segmental glomerulosclerosis with minimal interstitial fibrosis / tubular atrophy in biopsy also pointed to the acuity of IgAN. 

The renal pathology of IgA vasculitis (IgAV, formerly called Henoch-Schönlein purpura (HSP)) is IgAN. Naproxen was linked to IgA-mediated purpura fulminans but without delineating the kidney involvement. It was thought to be due to drug-induced protein C deficiency with hypocomplementemia [14]. Drug-induced hypersensitivity angiitis with granulomatous glomerulitis and interstitial nephritis was superimposed on pre-existing IgA nephropathy in a patient, though the authors could not determine which of the three drugs (piroxicam, aspirin, and bristocycline) was the culprit [15]. In 2 cases, piroxicam was linked to IgAV nephropathy but without renal biopsy [16]. Ibuprofen was associated with IgAV nephropathy in 1 case report [17]. Drug-related IgAV nephropathy was reported in a patient taking diclofenac, but the authors concluded it was caused by antibiotics instead of diclofenac [18]. Our case, to our knowledge, is the first report of NSAID-associated (specifically diclofenac) new-onset IgAN without systemic IgAV. Rofecoxib, a COX-2 inhibitor, was found to enhance serum IgA upregulation and mesangial IgA accumulation in an animal model of IgAN [19]. It will be interesting to know if this mechanism also applies to diclofenac which, like all other non-specific NSAIDs, is a COX-1 and COX-2 inhibitor. 

We acknowledge that more rigorous proof of a diclofenac-induced IgAN could not be performed in this case including repeat biopsy to demonstrate resolution of the IgA deposition and glomerulonephritis as well as re-exposure-associated disease recurrence. Thus, while our observations are strongly supportive, additional studies would be required to prove this novel association. 

In conclusion, we presented a case of AIN, MCD, and IgAN associated with diclofenac masquerading as a “herbal’ medicine. The cause was suggested by pathology and confirmed with high-resolution liquid chromatography mass spectrometry testing of the pills. A history of NSAID use should be diligently sought in any patient who presents with AIN and NS. This is the first report of IgAN possibly induced by NSAID without recurrence after 6 years’ follow-up. In addition, pills that may be listed as “herbal” may contain dangerous pharmaceuticals. Lack of regulation in the production process of these medications as well as limited user insight may result in potentially harmful and life threatening consequences. 

## Authors’ contributions 

All authors were directly or indirectly involved in the care of this patient. YC wrote the original manuscript. TP provided critical review and revision. PA helped testing the pill for its composition. JEZ provided pathological data and diagnosis. All authors revised and approved the final manuscript for publication. 

## Funding 

No funding was obtained for this study. 

## Conflict of interest 

The authors have no conflict of interest. 

**Table 1.** Laboratory values over time.

		**July 31, 2018**	**August 2, 2018**	**August 3, 2018**	**August 10, 2018**	**August 21, 2018**	**September 26, 2018**
Urine	Protein	> 500 mg/dL			3+	2+	Trace
	Blood	negative			2+	2+	Negative
	RBC	2/HPF			0-2	0-2	None
	WBC	7/HPF			0-5	6 – 10	None
	Pro/Cr (mg/g)	19, 040			7,410	1,372	190
	Hyaline cast	3/HPF			None	None	None
	Granular cast	3/HPF			None	None	None
Serum	BUN mg/dL	54	48	52	47	11	10
	Cr mg/dL	3.1	3.8	4.5	2.93	1.29	1.02
	Albumin g/dL		1.7	1.8	2.0	3.1	3.7
	Uric Acid mg/dL		8.9		5.1	4.0	7
	Chol mg/dL		346			254	215
	TG mg/dL		224			258	196
	HDL mg/dL		40			45	35
	LDL mg/dL		261			157	140

Cr = creatinine; BUN = blood urea nitrogen.

**Table 2.** Case reports of diclofenac-associated acute interstitial nephritis*.

**Case**	**Bio- ** **graphics**	**Latent period of diclofenac intake until onset of kidney disease**	**Clinical presentation**	**Renal biopsy**	**Interval from presentation to treatment**	**Treatment**	**Outcome**
Schwarz et al. 1988 [7]	54 yo male	Diclofenac for 6 months plus indomethacin for 7 weeks	AKI, peak Cr 4.98 mg/dL, proteinuria 0.1 – 0.5 g/d, Baseline Cr 1.13 mg/dL	Granulo-matous AIN	N/A	None	Renal function improved but remained CKD with Cr 2.26 mg/dL
Révai and Harmos 1999 [8]	65 yo female	6 months	AKI and NS	AIN, MN	Likely within days	Oral prednisone 1 mg/kg/d and tapering over 5 months	Complete remission of AKI and NS
Inoue et al. 2008 [9]	73 yo female	6 months	AKI and NS. Peak Cr 10 mg/dL Anuria requiring temporary hemodialysis, proteinuria 9.5 g/d	AIN, MCD	19 days	Oral prednisone 0.5 mg/kg/day tapering over 6 months	Complete remission of AKI and NS at 1 month after started on prednisone, Cr down to 1.1 mg/dL and proteinuria to 0.2 g/d
Akimoto et al. 2014 [10]	19 yo male	7 days but was discontinued 2 weeks prior to presentation for biopsy	AKI, peak Cr 2.55 mg/dL, proteinuria 0.51 g/d	AIN	15 days	Oral prednisolone 0.5 mg/kg/day tapering over 5 months	Complete remission of AKI, Cr down to 1.13 mg/dL 3 months later
Ozalp et al. 2021 [11]	44 yo male	Long-term use	AKI, baseline Cr 0.8 mg/dL 2 years prior, now peak Cr 5.2 mg/dL oliguria requiring temporary hemodialysis, Proteinuria 2.5 g/d	AIN	Likely within days	Oral methylprednisolone at a dose of 1 mg/Kg/day	Cr decreased to 2 mg/dL on day 10 of treatment and then tapered. But he continued to take diclofenac and 4 months later AKI again, peak Cr 9 mg/dL requiring HD again. Biospy showed CIN. Refractory to high dose steroid but after 4 months of HD, renal function better but remained having CKD with Cr 2.0 – 2.5 mg/dL
Oya et al. 2021 [12]	34 yo male	7 days but stopped when Cr rose, and 4 days later Cr peaked at 2.74 mg/dL	AKI and TINU, urine protein negative, baseline Cr 0.91 mg/dL	Granulo-matous AIN	N/A	Ophthalmic steroid only	Complete resolution of AKI and uveitis. Cr 1.29 1 month later and 1.03 3 months later
Our case	45 yo male	Intermittent use for ~ 1 year, with recent use for gout attack	AKI and NS, peak Cr 4.5 mg/dL, proteinuria 19 g/d.	AIN, ATI, MCD, IgAN	0 day.	Oral prednisone 0.8 mg/kg for total 9 days then 0.6 mg/kg for 2 days	Complete resolution of AKI and NS in ~ 1 month, Cr down to 1.02 mg/dL, without recurrence over 6 years’ follow-up.

AIN = acute interstitial nephritis; AKI = acute kidney injury; Cr = creatinine; CIN = chronic interstitial nephritis; TINU = tubulointerstial nephritis with uveitis syndrome; ATI = acute tubular injury; MCD = minimal change disease; IgAN = IgA nephropathy.
